# Impact of Dual Cell Co-culture and Cell-conditioned Media on Yield and Function of a Human Olfactory Cell Line for Regenerative Medicine

**DOI:** 10.3390/bioengineering7020037

**Published:** 2020-04-12

**Authors:** Rachael Wood, Pelin Durali, Ivan Wall

**Affiliations:** 1 Department of Biochemical Engineering, University College London, Torrington Place, London WC1E 7JE, UK; rachael.wood.14@ucl.ac.uk (R.W.); pelin.durali.14@alumni.ucl.ac.uk (P.D.); 2 School of Life & Health Sciences, Aston University, Aston Triangle, Birmingham B4 7ET, UK; 3 Institute of Tissue Regeneration Engineering (ITREN), Dankook University, Cheonan 31116, Korea

**Keywords:** olfactory ensheathing cells, spinal cord injury, neural regeneration, cell therapies

## Abstract

Olfactory ensheathing cells (OECs) are a promising candidate therapy for neuronal tissue repair. However, appropriate priming conditions to drive a regenerative phenotype are yet to be determined. We first assessed the effect of using a human fibroblast feeder layer and fibroblast conditioned media on primary rat olfactory mucosal cells (OMCs). We found that OMCs cultured on fibroblast feeders had greater expression of the key OEC marker p75NTR (25.1 ± 10.7 cells/mm^2^) compared with OMCs cultured on laminin (4.0 ± 0.8 cells/mm^2^, *p* = 0.001). However, the addition of fibroblast-conditioned media (CM) resulted in a significant increase in Thy1.1 (45.9 ± 9.0 cells/mm^2^ versus 12.5 ± 2.5 cells/mm^2^ on laminin, *p* = 0.006), an undesirable cell marker as it is regarded to be a marker of contaminating fibroblasts. A direct comparison between human feeders and GMP cell line Ms3T3 was then undertaken. Ms3T3 cells supported similar p75NTR levels (10.7 ± 5.3 cells/mm^2^) with significantly reduced Thy1.1 expression (4.8 ± 2.1 cells/mm^2^). Ms3T3 cells were used as feeder layers for human OECs to determine whether observations made in the rat model were conserved. Examination of the OEC phenotype (S100β expression and neurite outgrowth from NG108-15 cells) revealed that co-culture with fibroblast feeders had a negative effect on human OECs, contrary to observations of rat OECs. CM negatively affected rat and human OECs equally. When the best and worst conditions in terms of supporting S100β expression were used in NG108-15 neuron co-cultures, those with the highest S100β expression resulted in longer and more numerous neurites (22.8 ± 2.4 μm neurite length/neuron for laminin) compared with the lowest S100β expression (17.9 ± 1.1 μm for Ms3T3 feeders with CM). In conclusion, this work revealed that neither dual co-culture nor fibroblast-conditioned media support the regenerative OEC phenotype. In our case, a preliminary rat model was not predictive of human cell responses.

## 1. Introduction

Spinal cord injury (SCI) is a devastating injury to the central nervous system (CNS) that affects 250,000–500,000 new people worldwide every year. People with an SCI are 2–5 times more likely to die prematurely [1], and lifetime costs are estimated between US$1 and US$5 million, excluding indirect costs such as a loss in wages [2]. The majority of patients will suffer partial or complete paralysis [3] and can also suffer from chronic neuropathic pain syndromes, which have a serious effect on the quality of life [4]. Following SCI, inflammation and cell death ensue, and eventually, scar tissue forms, which is the main impediment to spontaneous regeneration, as the axons that do sprout and re-grow are unable to path-find through the scar to reach their target [5,6,7,8,9]. 

Unlike the rest of the body, the CNS does not regenerate. An exception is the olfactory system, which retains its ability to regenerate throughout adult life, due to the presence of a special type of glial cell, the olfactory ensheathing cell (OEC) [10]. These cells have been studied for potential use in spinal cord repair [11] due to their natural role in regenerating and guiding olfactory receptor neurons from the peripheral nervous system (PNS) into the CNS. Mucosal OECs can be biopsied via a minimally invasive intranasal approach, and although mucosa OECs tend to have lower yields and purity, they are more clinically attractive than their counterparts, bulb OECs, which are found in the lining of the brain [12]. 

Characterisation of OECs is commonly based on the expression of the neurotrophic receptor, p75NTR, in cell populations isolated from the olfactory mucosa or bulb [11,13,14,15]. p75NTR is a receptor that induces neurite outgrowth and cellular survival [16]; however, it is not a definitive marker for OECs [17]. In addition to p75NTR, glial cell marker S100β is commonly used as a positive marker for OECs [18], and Thy1.1 and fibronectin (Fn) are used as markers for contaminating fibroblasts [11,19,20,21]. 

For an OEC therapy to be developed, more understanding is needed of how to control the growth of functional subsets that express glial markers p75NTR and S100β and also how accurately these putative identity markers are predictive of function. If we better understand the markers that predict regenerative potential, we can then make strides toward enriching cells expressing those markers.

Due to the rapid onset of damage that occurs during an SCI, surgical options have to be enacted within 24 h of the injury [22], and by three weeks, the majority of the damage has occurred, so it is important to administer any cell therapy before this point [23]. A key issue with OECs is that their yield and purity variance cannot be predicted. Due to this, allogeneic cell therapy is seen as the most promising option as an off-the-shelf therapy that can be administered in the vital period before severe secondary damage sets in [24,25]. With the variability between patients, there would be no guarantee that enough functional cells could be produced in the required time frame, let alone all the release testing carried out to ensure the patient’s safety. Previously, our group generated conditionally immortalised human mucosa-derived OEC cell lines [26] toward the goal of producing an off-the-shelf therapy for SCI. In this study, we aim to compare our human cell line against primary rat cells to determine the best conditions for OEC culture and identify the relevance of animal studies in this area.

## 2. Materials and Methods

### 2.1. Primary Cell Isolation and Culture

Mucosae were isolated following the optimum protocol previously identified [27]. The mucosae were dissected from three adult female Sprague-Dawley 200–250g rats, which were euthanized by carbon dioxide asphyxiation (Schedule 1 method [28]) according to the U.K. Animals (Scientific Procedures) Act 1986. Each rat provided two mucosae. Each mucosa was placed in DMEM/F12 media to be transported to the laboratory. The mucosae were placed in Hanks’ Balanced Salt Solution (HBSS, Gibco Life Technologies, Gaithersburg, MD, USA) with 1% P/S (10,000 units penicillin, 10 mg streptomycin/mL) in a petri dish and washed by gently wiping each mucosa across a spatula. After washing, the mucosae were placed on a petri dish and cut up into small pieces, then placed in a 5 mL dispase II (2.4 units/mL, Sigma-Aldrich, UK) solution for 45 min at 37 °C in order to digest the tissue enzymatically. 

The mucosa and dispase solution was placed in the centrifuge for five minutes at 400× *g*. The supernatant was discarded, and the cells and tissue were re-suspended in 5 mL collagenase (Type I solution, 0.05%, Sigma-Aldrich, UK) for 15 min at 37 °C. Every five minutes, the collagenase was taken out of the incubator and mechanically triturated briefly before being placed back in the incubator. At the 15 minute point, the collagenase suspension was placed in the centrifuge for five minutes at 400× *g*. The cells were re-suspended in 7 mL DMEM/F12 media (2% FBS, 1% P/S) in a T25 flask at 37 °C in 5% CO_2_ for 24 h. All cell cultures were carried out at 37 °C and 5% CO_2_. 

The cells were placed in a tissue culture flask for 24 h as a differential adhesion step. The purpose of this step was to decrease the amount of contaminating fibroblasts in the culture. Fibroblasts have a faster adhesion time than OECs. After 24 h, most fibroblasts will adhere to the tissue culture plastic, whereas OECs will still be in suspension [27]. After 24 h, the suspension was replated onto laminin-coated (20 μg/mL, Sigma-Aldrich, UK) wells. The wells were coated for four hours at 37 °C. The laminin was then removed, and the cells were plated while the matrix was still wet. 

### 2.2. Cell Line Generation and Culture

The feeder layer (HuG418) was prepared from cells isolated from the human olfactory mucosa. The cells used were thawed from a pre-existing cell line prepared by Dr. Melanie Georgiou (UCL). The protocol followed by Dr. Melanie Georgiou was summarised in the paper by Pollock et al. [29]. The human mucosa OEC line was generated using the same technology; however, the purification steps were carried out before immortalisation to ensure a purer OEC population. The generation and culture conditions of this line (PA5) were covered by Santiago-Toledo et al. [26], and the culture conditions remained the same for HuG418. 

### 2.3. Feeder Layer Generation

Feeder layers were prepared by removing the media from the T75 flask and replacing with 4 mL mitomycin C (MMC) (Sigma-Aldrich, UK) for 2 h at 37 °C to inactivate the cells. After two hours, the MMC was removed from the cells, and the cells were washed with PBS (Lonza). The cells were removed from the flask using Trypsin/EDTA (Sigma) and placed in the centrifuge for five minutes at 400× *g*. After discarding the supernatant, cells were re-suspended in DMEM/F12 and plated up at 12 × 10^3^ cells/cm^2^. This density was used as it gave a good coverage of the well whilst allowing space for the OECs on the well surface. Feeders remained in the incubator for two days before any cells were plated on them. 

### 2.4. Collection of Conditioned Media

Conditioned media (CM) was collected from HuG418 cells after two days of culture. The media was centrifuged for five minutes at 400× *g*. CM was mixed at 1:1 with fresh media and added to the required conditions. 

### 2.5. Culture of PA5 Cells

For the experiments involving PA5 cells, wells were coated either with Ms3T3 feeders, poly-l-lysine (PLL), or laminin. PA5 cells were plated at 6000 cells/cm^2^ and cultured for five days. After five days, cells were fixed and stained for S100β and fibronectin. Neurotrophic factor 3 (NT-3) was added to some media conditions at 50 ng/mL as the literature indicated that NT-3 encourages the neurological repair phenotype in OECs [27]. 

### 2.6. In Vitro Co-culture with NG108-15 Neurons

NG108-15 neurons were either grown alone on Ms3T3 feeders (as a control) or on a layer of PA5 cells that were seeded onto either laminin or Ms3T3 feeders. NG108-15 neurons were seeded at 500 cells/cm^2^ onto the laminin-coated plates or feeder layers. Co-culture was carried out for five days with a media change occurring on Day 3 following the protocol laid out by Santiago-Toledo et al. [26]. Cells were fixed with 4% PFA at room temperature (RT), and cells were stained to detect β-III tubulin. 

### 2.7. Immunocytochemistry (ICC)

Cells were fixed with 4% paraformaldehyde (PFA, Sigma-Aldrich, UK) for 20 min at RT. The PFA was removed, and the cells were washed three times with PBS. Three washes with PBS were carried out in between every step of the immuno-staining in order to remove and dilute any traces of the previous reagent. One wash was defined as filling the well with PBS and leaving for five minutes. Zero-point-two-five percent Triton-X (Sigma-Aldrich, UK) was added to each well and left for 20 min at RT. All solutions were made up in PBS. Five percent goat serum solution (Dako) was used as the blocking solution for 30 min at RT. Primary antibodies were prepared at a dilution of 1:200 and added for 90 min at RT. The primary antibodies used were as follows: rabbit anti-p75NTR (Millipore), mouse anti-Thy1.1 (Millipore), rabbit anti-S100β (Dako), mouse anti-fibronectin (Sigma-Aldrich, UK), and mouse anti-βIII tubulin (Sigma-Aldrich, UK). The negative control was subjected to the same treatment as other wells, the exception being the exclusion of the primary antibody. The secondary antibodies (DyLight^®^ 488 or 594, goat anti-rabbit IgG (H + L) and goat anti-mouse IgG (H + L), Vector Laboratories, Burlingame, CA, USA) were prepared at 1:200 and Hoechst (Sigma-Aldrich, UK) at 1:1000 and incubated for 45 min at RT. Fluorescent imaging was carried out on an EVOS FL microscope (Life Technologies). 

Five images were taken per well and were taken in a cross formation around the centre of the well. Two to three wells were stained per condition. After imaging, each channel was examined to assess whether the images taken were representative of the well. Brightness and shutter speed were set by identifying a cell that was determined to be positive and ensuring the background was dark to prevent overexposure of the cells. These settings were held constant between images for an experiment to ensure the images could be directly compared. 

Images were analysed using ImageJ. A colour threshold was set to identify a positive cell, and only cells that were identified to be above this threshold were counted as positive. Positive cells were counted, and the results were calculated as the proportion of cells positive for the marker and the yield of positive cells over the imaged area. 

### 2.8. Circularity Analysis

A macro was written in ImageJ in order to analyse the morphology of the cells. Circularity was used as the defining value. The macro was written in JavaScript to automate a calculation method that already exists in ImageJ. The “adjust threshold” window opens automatically when the macro is run and can be adjusted to select the area of interest. Any cell that was larger than a set threshold (this value can be adjusted depending on whether the nucleus or the whole cell is being analysed) was discarded to prevent cell clusters being included in the calculation. In addition to this, any cells on the boundary of the image were discarded so they did not contribute to false readings. The circularity was calculated as in Equation (1), where *A* is the area (pixels^2^) and P is the perimeter (pixels).

(1) $$Circularity=\frac{4A\pi }{P^{2}}$$

A circularity of 1 indicates a perfect circle, and a circularity of 0 indicates a straight line. When the macro was run, a printout of the circularities was displayed. These data were exported to GraphPad^®^ and plotted as a histogram.

### 2.9. Neuronal Growth Analysis

According to the literature, five days of co-culture are optimal for observing enhanced neurite outgrowth compared with three days of co-culture [27]. After five days of co-culture, cells were labelled with antibodies against βIII-tubulin. Neuron number, neurite number, and neurite length were quantified using the NeuronJ plugin in ImageJ [30]. Neurites were traced and measured within the software and exported to GraphPad^®^ for analysis. 

### 2.10. Statistical Analysis and Data Accessibility 

Data are presented as the mean ± standard error of the mean (SEM). For the majority of the data, one-way ANOVA was used to determine statistically significant differences, and the Bonferroni correction was carried out using GraphPad^®^ software to calculate *p*-values. When there were only two sets of data to compare, the Shapiro–Wilk test was used to test for normality. When data were normally distributed, the Tukey test was used to test for significance. When data were not normally distributed, the Kruskal–Wallis test was used. The Bonferroni correction was not used for sets of data that only compared two conditions as there was no need to correct for multiple comparisons. For the comparison of histograms, the Kolmogorov–Smirnov test was carried out. 

In this work, one experimental repeat (n) was defined as cells taken from different flasks/groups of animals. 

Statistics are only reported in the text when there are significant differences at α = 0.05. In all figures, a single asterisk (*) indicates *p* < 0.05, two asterisks (**) *p* < 0.01, and three asterisks (***) *p* < 0.001.

The datasets generated and analysed during this study are available from the corresponding author upon reasonable request. 

## 3. Results and Discussion

### 3.1. Human Feeders Encourage an Increase in p75NTR and Spindle-Shaped Cells in Rat OECs

**Figure 1:**
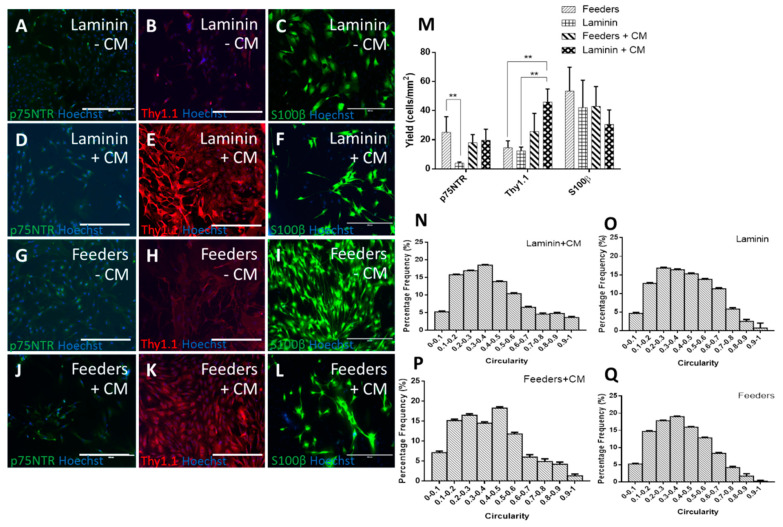
Fluorescent micrographs of primary rat olfactory mucosal cells (OMCs) cultured on laminin (**A–F**) and HuG418 feeders (**G–L**) in the presence (**D–F**, **J–L**) and absence (**A–C**, **G–I**) of HuG418 conditioned media and stained for olfactory ensheathing cell (OEC) biomarkers p75NTR and S100β and fibroblast marker Thy1.1. Positive cells were counted in ImageJ and calculated as the number of positive cells over the image area (**M**). OMCs cultured on laminin with standard media had the lowest yield for p75NTR, and the addition of conditioned media caused an upregulation of undesirable marker Thy1.1. Circularity was used as a measurement of morphology, and positive S100β cells were analysed for their circularity (**N–Q**). Cells on laminin with standard media and feeders with conditioned media gave more enlarged cell morphologies. The scale bars represent 400 µm. Data are the means ± SEM., n = 3. CM, conditioned media.

The ICC was quantified using yield (positive cells per mm^2^), but not purity, as the presence of the feeders in only some conditions would make any assessment of purity misleading. It can be observed from Figure 1B,E,H,K,M that the addition of CM significantly increased the expression of Thy1.1. When CM was added to OECs cultured on feeders, an increase in Thy1.1 over and above the higher expression induced by feeders with standard media was observed (25.7 ± 12.4 cells/mm^2^ on feeders with CM from 14.5 ± 4.8 cells/mm^2^ on feeders with standard media). The increase in Thy1.1 expression observed on laminin with CM (45.9 ± 9.0 cells/mm^2^) indicated that HuG418-derived CM affected Thy1.1 expression more when the feeders themselves were not present. This could be due to the feeders supporting themselves. Where feeders were present, those cells could uptake some of the soluble factors present in the CM, leaving a lower concentration of soluble factors for the OECs. This would mean that there were more soluble factors in the media when the feeders were not present. Fibroblasts participate in paracrine signalling [31,32], and therefore, it follows that if they are not present to receive the factors, the factors present can significantly assist other cells present.

p75NTR was present in all conditions at a similar level, with the exception of laminin with standard media, which was significantly lower at 4.0 ± 0.8 cells/mm^2^. The presence of CM increased the expression of p75NTR on laminin (19.7 ± 7.6 cells/mm^2^). An increase in p75NTR expression without the presence of the feeders would imply that there was some form of paracrine signalling occurring. Whether this was due to FGF2 expression or a combination of other factors would require a deeper examination of the components in the CM. Without the higher expression of Thy1.1, CM with laminin would be a promising condition to take forward. Thy1.1, historically an undesirable marker of fibroblast phenotype in the OEC field, negated the positive effect of the increase in putative OEC marker p75NTR as it implied a higher level of fibroblast impurities that would need to be removed from the culture. 

The morphology of S100β-positive cells was analysed using an automated circularity macro in ImageJ (Figure 1N–Q). Numerical analysis revealed that there was no significant difference between the distributions for feeders versus feeders with CM, and both conditions had a left skew (skew = 0.4 and 0.5, respectively). The laminin condition had a skew value of 0.3 and a kurtosis value of −0.8. When CM was added to the laminin condition, the distribution was significantly different from the standard laminin condition (Kolmogorov–Smirnov, *p* = 0.01). The distribution with CM had a less flattened distribution (kurt = −0.3 compared to −0.8) and a more pronounced left shift (skew = 0.7 compared to 0.3). This was a stronger shift than the feeder conditions. This was significant as this was the condition with the most Thy1.1-positive cells. The most likely option was that spindle-shaped OECs were also expressing Thy1.1, as well as S100β. Co-labelling of these antibodies was not carried out in this study due to the lack of consistency experienced with the separate S100β antibody, which meant the Thy1.1 and S100β antibodies used were raised in the same species. The literature indicated that it is possible that OECs do co-express p75NTR and Thy1.1 [33,34,35,36] and Thy1.1 and GFAP [34], so the assumption that Thy1.1 is a contaminating cell marker may need to be re-examined. Due to the lack of putative OEC markers [37,38], these cells are poorly characterised, and this presents one of the major challenges of OEC research. 

**Figure 2:**
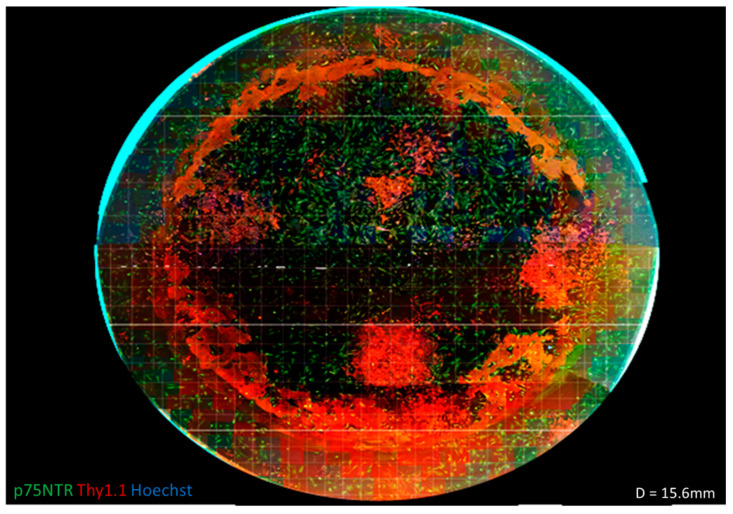
Assembled fluorescent micrograph of a cell population derived from rat olfactory mucosa cultured on a human feeder layer of olfactory fibroblasts. Images were taken at 100× total magnification in a 24 well plate (diameter = 15.6 mm) and stitched together using PanoramaPro2. It can be seen from this stitched image that there are definite populations present in the well plate. There are distinct areas of Thy1.1 (red) that do not appear to be dispersed with the areas of p75NTR (green). This variety in present populations explains the large error bars found in the cell counts. When multiple views were taken for counting, the purity could range from 0–100%, which relates to a large standard deviation.

Although a differential adhesion step was used to remove rapidly adherent cells, leaving the slow attaching OECs in culture, the primary culture could still harbour contaminating cell types. This is demonstrated in Figure 2, where an entire well of OECs on feeders was imaged at 10× objective magnification (EVOS Life Technologies AMF4300) and the resulting images merged together using PanoramaPro2 in order to gain an understanding of the culture as a whole. It was observed that there were distinct areas of Thy1.1-positive colonies. When images were taken for counting, these images could yield data from 100% p75NTR-positive to 0% p75NTR-positive. This wide range of values resulted in a large standard deviation and therefore a large standard error of the mean across independent experiments (seen Figure 1M). These large errors were due to stochastic variability in the cultures due to the different cell subsets present, and these different cells could predominate in different regions of a single dish. From a cell manufacturing perspective, this created a significant challenge for creating a robust and well-characterised product.

After staining, there was also a red/yellow ring around the periphery of the plate that was not associated with any positive cells. On close inspection of the well plates used, it was found that the bottom of the wells was rarely smooth, and often, there were circular scratches, presumably as a result of the manufacturing process. It was postulated that the antibody interacted with this non-smooth surface or was trapped in ECM deposited as a consequence of cell responses to the surface scratches and gave the circular staining [39]. This pattern was observed in several images; however, it was not until the full well was imaged that it was noticed that it made a complete circle. 

### 3.2. Comparison of Different Feeder Layers

**Figure 3:**
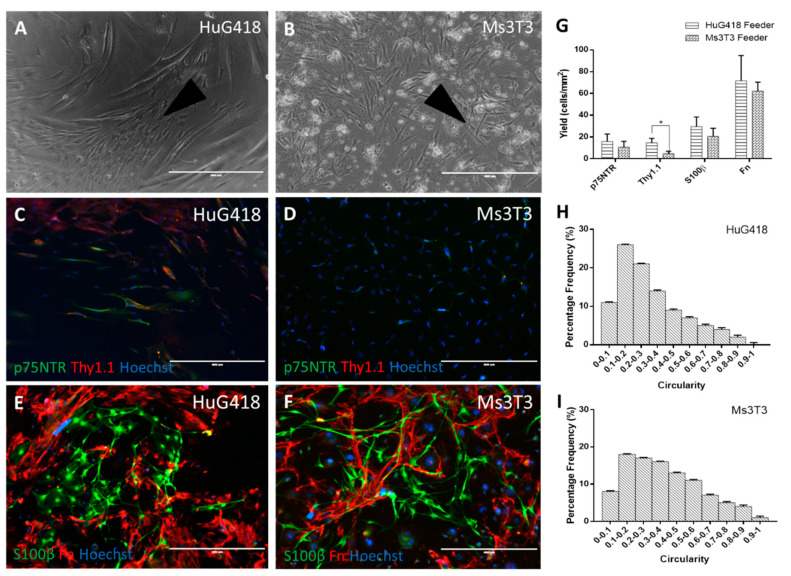
Cells were fixed after 14 days of culture and stained for OEC biomarkers p75NTR and S100β and fibroblast biomarkers Thy1.1 and Fn (**A–D**). Positive cells were quantified in ImageJ and graphed as the yield of positive cells in the image area (**E–G**). Circularity was used as a measurement of morphology, and positive S100β cells were analysed for their circularity (**H**,**I**). The scale bars represent 400 µm. Data are the means ± SEM, n = 3.

One striking observation in the S100β (detecting OECs) and Fn (fibroblasts) staining condition comparing Ms3T3 and HuG418 feeders (Figure 3) was that the OECs did not appear to grow directly over the feeder layer. Instead, they grew close to the fibroblasts (Figure 3C,D). This placement made sense if the cells were gaining benefits from cell-to-cell contact. If paracrine factors were involved, the OECs would not necessarily border the fibroblasts so closely [40]. 

No differences in p75NTR expression were observed on the different feeder layers (Figure 3A,B,G) with yields of 15.9 ± 6.8 cells/mm^2^ and 10.7 ± 5.3 cells/mm^2^ on Ms3T3 and HuG418, respectively. This may indicate that the OECs were benefitting from general cell-to-cell contact, not specifically anatomically-matched HuG418 cells. However, as both lines were comprised of fibroblasts, there could also be a common paracrine factor involved. OECs have been well documented to grow poorly in isolation, and when a low yield is obtained, they do not proliferate [41,42,43]. Additionally, they have been observed to survive better in a culture with a mixed population (normally OECs and olfactory fibroblasts) [10,44,45,46,47]. This would also explain why the HuG418 CM did not have the anticipated beneficial impact on OECs’ p75TNR expression as hoped as the cells themselves may be required. In the literature, it has been observed that there is a close relationship between OECs and olfactory fibroblasts. However, it does appear that this relationship is due to the physical contact as opposed to any paracrine factors present [48]. 

Thy1.1 expression was found to be significantly different between the two feeders (Kruskal–Wallis, *p* = 0.01). As observed in the ICC images and quantification, the presence of Ms3T3 downregulated the expression of Thy1.1 (yield of 14.6 ± 4.1 cells/mm^2^ on HuG418 and 4.8 ± 2.1 cells/mm^2^ on Ms3T3). This was important as it indicated that the Ms3T3 feeders were superior to the HuG418 when it came to supporting OEC growth and expression of p75NTR. OECs appeared to be supported by cell-to-cell contact and did not gain any benefit from paracrine factors. These results suggested that Ms3T3 feeders were superior to HuG418 cells as they resulted in fewer Thy1.1-positive cells whilst maintaining similar p75NTR expression levels. 

These results, together with the results obtained with CM, indicated that OECs benefitted from cell-to-cell contact rather than paracrine signalling by HuG418 cells. Ms3T3 cells are commercially available as a GMP cell line (Sigma-Aldrich, UK). Therefore, their application as a feeder layer to enhance OEC phenotype during scalable manufacture is attractive to advance OECs towards the clinic. Potential safety concerns around the purity of the final clinical preparation could be addressed by purifying OECs with affinity-based removal of the mouse feeders. From this initial work with primary rat OECs, it was found that rat OECs have a more promising regenerative function when cultured with feeders. However, there was no difference between human olfactory mucosa-derived feeders and mouse 3T3 cells in their ability to support OEC marker expression. 

### 3.3. Human OECs Behave Differently to Rat OECs in Culture 

Studies in small animals are important to generate pre-clinical data and to gain an understanding of responses in physiologic systems before moving into humans. However, there are concerns that OECs in different animals behave differently [49,50,51], and animal cells and tissue do not accurately reflect the function or behaviour of human material. This is especially important when the length of the injury is considered. The gap to be bridged by the neurons in an SCI is much smaller in a rat compared to a human [50], and the ability of OECs to support neurons over this small distance may not be relevant on the human scale. Therefore, we generated conditionally immortalised cell lines from the human olfactory mucosa [26] and tested these under the same conditions in order to determine how translatable animal studies are. 

**Figure 4:**
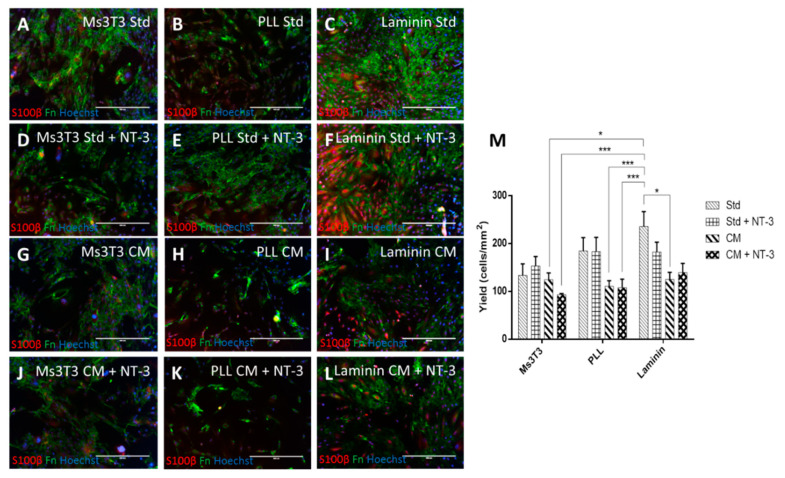
PA5 cells cultured on laminin or Ms3T3 feeders, in standard media or HuG418 conditioned media. Cells were cultured for five days and stained for S100β and Fn (**A–L**). S100β-positive cells were quantified in ImageJ and graphed as yield of positive cells in the image area (**M**). Conditioned media constantly gave lower yields of S100β, and laminin with standard media gave the highest yield. The scale bars represent 400µm. Data are the means ± SEM, n = 3.

ICC micrographs in Figure 4 revealed that Fn staining on laminin occurred in a completely different pattern from the other matrix conditions. In Ms3T3 and PLL conditions, the Fn deposition could be more easily traced back to the individual cells producing it. In contrast, on laminin, the staining pattern was more widely distributed. These distinct differences in the pattern were unexpected. Due to this staining pattern, cell counts and yield were not carried out for Fn due to the inaccuracy of determining the original cell responsible for the Fn. The reason for the different staining pattern on laminin is not known, and no references to similar occurrences could be found in the literature. 

S100β expression appeared to be more prominent in conditions with NT-3 compared with those conditions cultured in its absence and CM did not have any positive impact on S100β expression (Figure 4). From the three matrix conditions, it was found that the presence of Ms3T3 feeders was less beneficial for glial marker expression compared with PLL and laminin, which contrasted what was observed for rat OECs (yield of 133.7 ± 24.0 cells/mm^2^ on Ms3T3 with standard media compared to 184.8 ± 27.7 cells/mm^2^ and 235.9 ± 31.0 cells/mm^2^ for PLL and laminin with standard media, respectively). This was not necessarily surprising, as it has been found that there are several differences between the rat and human olfactory system [52] including in vitro growth, spontaneous immortalisation, and morphology [53,54]. Additionally, in terms of implantation and isolation, the olfactory mucosa in rats is yellow, whereas in humans, it cannot be discerned from the respiratory tissue [55]. This leads to a higher level of non-OECs being present in the implant, which may or may not assist in regeneration. It does cause concern that any previous work that has been carried out in rat is not translatable to human scale up [24,49]. 

Another difference between the rat and human OECs is that the human OECs do not appear to benefit from the presence of NT-3. Although the study was not carried out as part of this work, previous studies have shown that NT-3 significantly increased the expression of glial markers in rat cells [27]. For our human cell line, the presence of NT-3 seemed to increase the level of expression slightly, but not significantly. 

The differences that occurred between rat and human OECs in regard to neurotrophic factors and matrix preference showed that more in depth study and understanding of human OECs are necessary to be able to predict cell behaviour. It also indicated that care needs to be taken when translating results from rat to human OECs. Two human cell lines were investigated (data not shown from the second cell line), and they both followed the same pattern with laminin being the highest performing condition in terms of yield and significantly higher than most of the conditions with CM. Due to this, human OECs were carried forward into the future work to ensure the conditions investigated were relevant to the clinic. 

### 3.4. Human OEC Co-culture with Neurons

Neurite outgrowth in cultured neurons is considered an indication of neuro-regenerative potential [56,57,58,59,60], and therefore, neurite outgrowth was quantified to compare the cultures. Neurite number and length were normalised to the number of neurites and neurons. Neurons were cultured in isolation to ensure any neurite extension we observed could be related to the presence of the OECs. 

**Figure 5:**
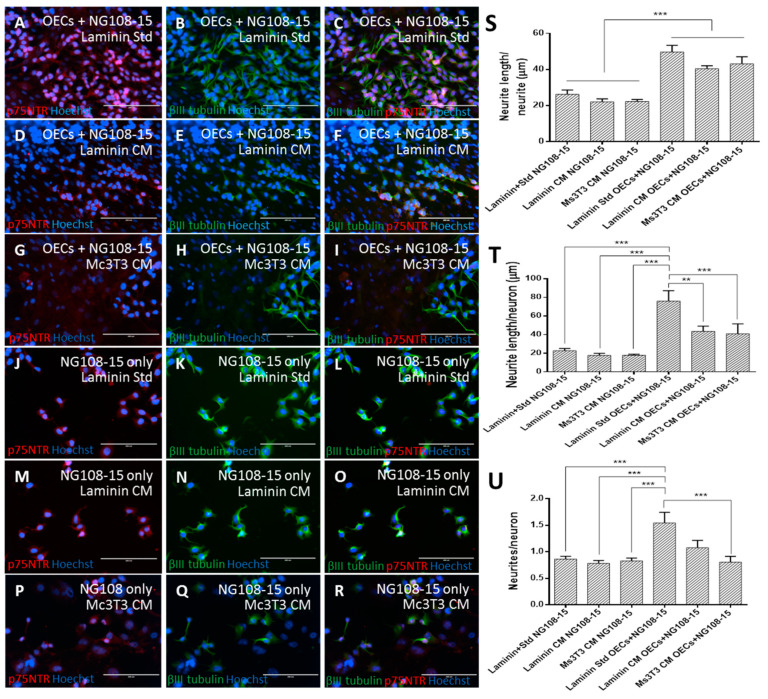
PA5 cells were co-cultured with NG108-15 neurons for five days under different matrix and media conditions. NG108-15 cells were also cultured in isolation to ensure that any improvement observed could be reliably attributed to the PA5 cells as opposed to the matrix and media conditions. After five days of NG108-15 culture, cells were fixed and stained for βIII-tubulin and p75NTR (**A–R**). The NeuronJ plugin in for Image J was used to quantify the neurite outgrowth, and measurements were made for neurite length/neurite (**S**), neurite length/neuron (**T**), and neurites/neurons (**U**). The scale bars represent 200 µm. Data are the means ± SEM, n = 3.

Cells co-cultured on laminin with standard media had a higher number of neurites with longer extensions (Figure 5). Cells co-cultured on laminin and feeders with CM did not perform as well as cells cultured with standard media. No difference was observed between the neuron-only conditions on the different matrices for all measurements. This indicated that the neurons were responding to the PA5s as opposed to the culture conditions (matrix and media). 

The conditions cultured with OECs performed best in regard to average neurite length (Figure 5S, one-way ANOVA, Bonferroni post-hoc, *p* < 0.001). Although co-culture on laminin with standard media performed better than those co-cultured with CM (49.7 ± 3.7μm versus 40.4 ± 1.6 μm), this did not result in any significant difference. 

When the neurite length per neuron was examined (Figure 5T), the co-culture on laminin with standard media showed significantly longer neurites per neuron (76.0 ± 11.2 μm) compared with every other condition (43.6 ± 5.5 μm on laminin with CM and 41.0 ± 10.6 μm on Ms3T3 with CM, one-way ANOVA, Bonferroni post-hoc, *p* < 0.01). All conditions without OECs performed similarly to each other (22.8 ± 2.4 μm, 17.8 ± 2.3 μm and 17.9 ± 1.1 μm on laminin with standard media, laminin with CM and Ms3T3 with CM, respectively). 

The number of neurites extended by each neuron revealed a similar pattern (Figure 5U) where the co-culture on laminin with standard media produced more neurites per neuron (1.54 ± 0.20) than the majority of the other conditions (one-way ANOVA, Bonferroni post-hoc, *p* < 0.001) with the exception of co-culture on laminin with CM (1.08 ± 0.14). Collectively, these data showed that the matrix and media condition that gave the highest S100β expression in OECs resulted in the best neuronal growth support. 

The formation of neurites from neurons is vital to the functionality and development of the nervous system [61]. From these results, it can be seen that co-culture on laminin with standard media was the condition that provided the most support to the development of neurons in regard to the longest average neurite. In the absence of co-culture, the development of neurons was not well supported, and as a result, shorter neurites were observed. Previously, it was found that laminin with standard media was a promising condition for OEC proliferation and S100β expression. This suggested that when OECs were expressing higher levels of S100β and that they were able to provide better support to neurons. When S100β expression was lower, the presence of the OECs was still able to benefit the neurons. 

When Schwann cells support peripheral nerve repair, it has been observed that an upregulation of S100β leads to identification of so-called “reactive” Schwann cells, which are responsible for axonal sprouting [62]. If OECs followed a similar behavioural pattern, it could be that the conditions with higher S100β were more capable of allowing axonal sprouting and therefore resulted in increased neurite numbers. 

It has been observed in several studies that laminin is an extracellular matrix that is able to stimulate rapid neurite growth and has been directly linked to neurite outgrowth in vitro [63,64,65]. Studies have also shown that enhanced neurite outgrowth and preferred attachment was observed when neurons from the CNS were plated on laminin [66], and it is thought that the presence of laminin starts to create a more permissible environment for axonal extension [67]. The hostile environment present after SCI is a key part of why nerve regeneration does not happen spontaneously [24,68,69]. There was no significant difference observed between neurons cultured in isolation on laminin and on Ms3T3 feeders. This suggests there is more to the interaction than the preference for laminin. The combined effect of the favourable matrix and support cells could explain why laminin with standard media was the highest performing condition. 

CM collected from HuG418 had a lesser effect on the average neurite length per neuron compared to standard media. This may be initially related to the lower S100β expression in the OECs in this condition. It would indicate that the CM from HuG418 does not have any beneficial soluble paracrine factors for the neurons. This lack of factors and therefore interaction between these two populations is not necessarily unexpected as although studies have shown a benefit in transplantation with a mixed population of OECs and fibroblasts (Keyvan-Fouladi et al., 2003 [44], Ramón-Cueto et al., 2000 [10], Raisman and Li, 2007 [47], Teng et al., 2008 [46]), it has been under the understanding that the fibroblasts support the OECs, not the neurons. Fibroblasts have not been pursued as a cell therapy option for nerve regeneration, and they are not believed to have specific properties that enhance the function of neurons [70,71]. Therefore, standard media resulting in longer neurites than conditioned media can be seen as an expected output. 

## 4. Conclusions

We established that the behaviour of rat and human OECs did not follow the same patterns, and therefore, caution needs to be engaged when using pre-clinical data that have been carried out using rat models. When focusing on human OECs, we found that out of the conditions investigated, laminin with NT-3 was the best condition for protein expression and neurite extension. It would be valuable to investigate the components of the fibroblast conditioned media to further understand what aspects resulted in Thy1 upregulation and how the population reacts when Thy1 positive cells are removed.
